# Supplementary material for: Identification of immunogenic cell death signature genes in hepatocellular carcinoma: from single-cell transcriptomics to in vitro mechanistic validation and comprehensive prognostic modeling with hundreds of machine learning algorithms
Source: Front Immunol. 2025 Oct 22;16:1649618. doi: 10.3389/fimmu.2025.1649618 (PMC12585982; doi:10.3389/fimmu.2025.1649618)
Supplement: Supplementary file 1 [file DataSheet1.docx]

Supplementary Material

#
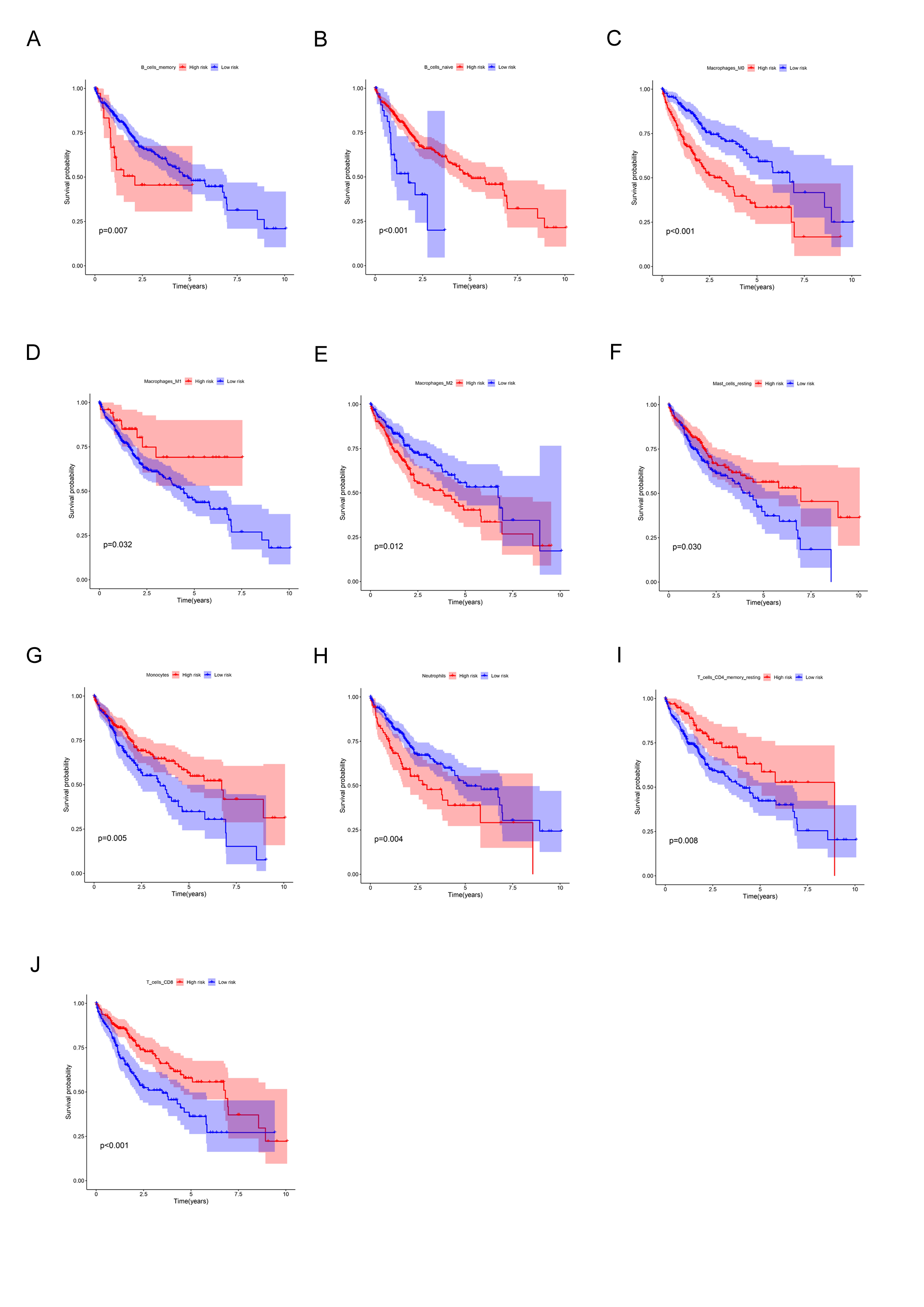
Supplementary Figure

**Supplementary Figure 1:** Kaplan-Meier survival analysis of individual immune cell types in HCC patients. (A) B cells memory; (B) B cells naive; (C) Macrophages M0; (D) Macrophages M1; (E) Macrophages M2; (F) Mast cells resting; (G) Neutrophils; (H) Neutrophils; (I) T cells CD8 memory resting; (J) T cells CD4. P-values were calculated using the log-rank test. Only immune cell types with significant associations (p<0.05) with overall survival are shown.
